# Functional brain activation changes associated with practice in delaying smoking among moderate to heavy smokers: study protocol and rationale of a randomized trial (COPE)

**DOI:** 10.1186/s13063-018-2984-x

**Published:** 2018-11-12

**Authors:** Andrew T. Fox, Delwyn Catley, Kimber P. Richter, Edward F. Ellerbeck, Morgan G. Brucks, Vlad B. Papa, Laura E. Martin

**Affiliations:** 1 Hoglund Brain Imaging Center, University of Kansas Medical Center, Mail Stop 1052, 3901 Rainbow Blvd, Kansas City, KS 66160 USA; 2 Center for Children’s Healthy Lifestyles and Nutrition, Children’s Mercy Kansas City, 2401 Gillham Rd, Kansas City, MO 64108 USA; 3 Department of Preventive Medicine and Public Health, University of Kansas Medical Center, 3901 Rainbow Blvd, Kansas City, KS 66160 USA

**Keywords:** Smoking, Functional magnetic resonance imaging, Randomized trial, Study protocol

## Abstract

**Background:**

Most smokers struggle to overcome tobacco addiction. Neuroscientific models of addiction emphasize the importance of brain regions associated with cognitive control and reward to understand the cycle of addiction and relapse. During an attempt at abstinence, the cognitive control system appears to be underpowered to override the heightened reward system of the addicted brain. Thus, one neural target for treatment is to strengthen the cognitive control system. It may be possible to improve the functioning of the cognitive control system via deliberate practice.

**Methods/design:**

This study will determine the effects of practicing delaying smoking on brain and behavioral measures of cognitive control. Smoking patterns will be monitored for 1 week and then smokers (*N* = 80) will be randomized to either practice cognitive control by delaying their first cigarette of the day for 2 weeks (practice group) or they will continue monitoring only (no practice group). Functional magnetic resonance imaging will be performed while smokers regulate their responses to smoking images (i) at baseline and (ii) after 2 weeks of practice (or no practice).

**Discussion:**

The primary aim of this study will be to identify the impact of practicing cognitive control on functional brain activation changes in response to smoking cues. If successful, this project will establish a neurobiological biomarker for increasing cognitive control and demonstrate the feasibility of neuroimaging methods to predict the efficacy of an intervention without a large clinical trial.

**Trial Registration:**

ClinicalTrials.gov, NCT03080844. Registered March 15, 2017

**Electronic supplementary material:**

The online version of this article (10.1186/s13063-018-2984-x) contains supplementary material, which is available to authorized users.

## Background

Thirty-six million American adults smoke cigarettes [1] despite the known negative consequences [2]: nicotine addiction is difficult to overcome. Dual-system models of addiction assert primary roles for two systems: the cognitive control network (dorsolateral (dlPFC), dorsomedial, and ventrolateral prefrontal cortices [3–5]) and the reward network (ventromedial (vmPFC) and medial prefrontal cortices, and striatal regions [3–5]). In addiction, the cognitive control network is considered underactive while the reward network is considered overactive [3–6], resulting in a reward network that overrides the cognitive control network leading to relapse during quit attempts [4, 5, 7, 8].

An important implication of these models is that pharmacotherapy and/or behavioral treatments for smoking cessation should ideally target both systems to improve outcomes [4, 5]. There is evidence that bupropion and varenicline reduce activation in the reward network [9–12], but there is little evidence for any interventions that impact the cognitive control network. Cognitive-behavioral smoking cessation treatment commonly involves identifying and altering smoking cues (triggers), self-monitoring, and cognitive restructuring [13]. In clinical practice, smokers are encouraged to develop a quit plan whereby they self-administer these techniques to resist cravings and maintain abstinence [13, 14]. Counseling patients to use these techniques after they quit significantly improves cessation rates [15]; however, it is unclear whether using these techniques impacts either the cognitive control or reward networks.

Although the neural impact of behavioral cessation treatment is unknown, evidence from research on cognitive behavioral therapy (CBT) for depression indicates that practicing behavioral strategies can impact cognitive control areas [16, 17]. This suggests that cognitive control *practice* could enhance smokers’ ability to resist cravings and improve success at maintaining abstinence. If true, this would have important treatment implications because current behavioral treatment does not routinely implement practicing behavioral strategies *prior* to making a quit attempt. Emphasizing cognitive control practice prior to quitting could theoretically improve the effectiveness of cessation by enhancing smokers’ cognitive control capacity.

The purpose of this study is to examine among smokers the effect of cognitive control practice on cognitive control brain activation during efforts to regulate craving. Brain activation can be measured non-invasively using functional magnetic resonance imaging (fMRI) while smokers view smoking cues or images that trigger cravings [18–21]. Greater efforts to regulate craving (i.e., exerting cognitive control) have been linked to greater activation of the dlPFC [15], which is implicated in the cognitive control network [14]. In this study, participants will be randomized to 2 weeks of practice in exerting cognitive control by delaying the first cigarette of the day or smoking as usual; the comparison group will monitor their smoking behavior but will not be asked to delay smoking cigarettes. This study will identify whether: (i) cognitive control practice leads to changes in brain activation, (ii) associations exist between the amount of practice and brain activation changes, and (iii) individual differences in practice relate to brain activation changes. This will enhance our understanding of treatment mechanisms and provide a model for studying the impact of behavioral interventions on brain function.

### Study objectives

#### Primary objective

The primary objective is to identify the impact of practicing compared to not practicing delaying smoking on functional brain activation changes in the cognitive control networks in response to smoking cues. We hypothesize that practice vs. no practice will lead to greater brain activation increases in the cognitive control network when comparing pre- to post-intervention.

#### Secondary objective

In the group practicing delaying smoking, the secondary objective is to examine the association between the amount of practice (i.e., the amount of time the first cigarette of the day is delayed) and functional brain activation changes in the cognitive control network in response to smoking cues. We hypothesize that greater levels of practice will correspond to greater brain activation increases in the cognitive control network when comparing pre- to post-intervention.

#### Other objectives

Additional objectives are to examine the association between practice-related brain activation changes in the cognitive control network and individual differences (e.g., nicotine dependence, motivation, and sex). We hypothesize that practice-related increases in brain activation in the cognitive control network are expected to be greatest among more dependent and less motivated smokers as well as women.

## Methods/design

### Study overview

**Fig. 1:**
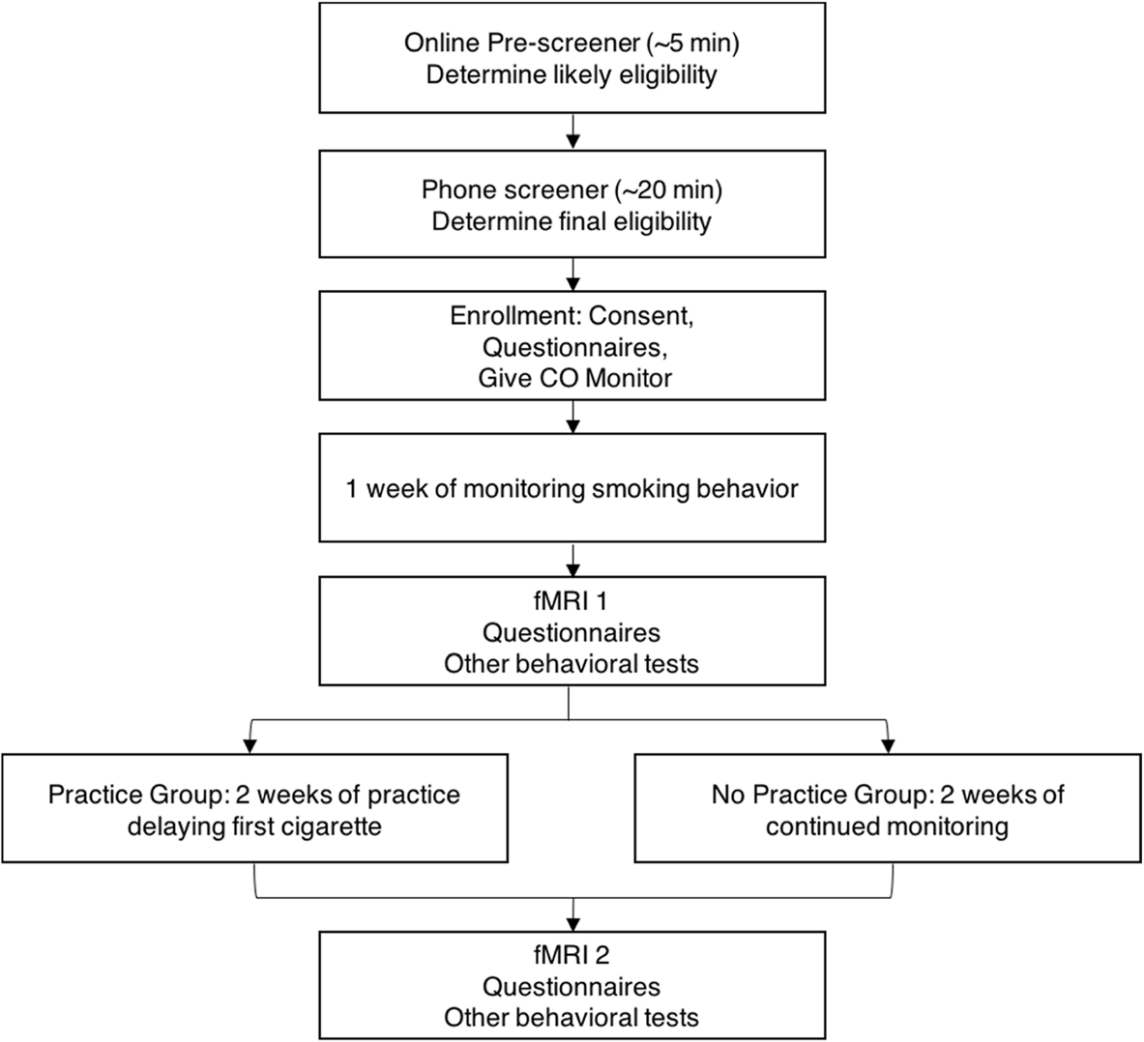
Flow chart of enrollment, interventions, and assessments

We will use a randomized design (parallel group, two-arm, superiority trial with a 1:1 allocation ratio) to examine the impact of practicing delaying the first cigarette of the day on cognitive control brain networks during an fMRI cue-reactivity task (see Fig. 1). Smokers (*N* = 80) will receive tips on how to resist the urge and control cravings to smoke and then be randomized either to practice progressively delaying their first cigarette of the day over a 2-week period (practice) or not to practice delaying their first cigarette and smoke as usual over a 2-week period (no practice). Portable carbon monoxide (CO) monitors will be used to assess objectively the timing of the first and second cigarettes of the day (days 1–7) and subsequently to monitor practice adherence (i.e., the delay of the first cigarette of the day; days 8–21). All participants will be given brief advice on techniques to help them control urges to smoke based on cognitive behavioral therapy (CBT) techniques. Participants will continue monitoring their smoking behavior regardless of group assignment. At baseline (day 7) and following 2 weeks of practice (day 21), participants in both arms will complete an fMRI session during which they view smoking and food cues and are asked to rate how much they want them. All study procedures will be conducted in accordance with the Code of Ethics of the World Medical Association (Declaration of Helsinki) and have been approved by the Institutional Review Board of the University of Kansas (protocol STUDY00004095). This experiment is registered at ClinicalTrials.gov (NCT03080844).

### Design justification

Our reason for delaying the first cigarette of the day as a way to operationalize practice is based on theoretical and practical considerations. The first cigarette of the day is typically one of the most preferred cigarettes of the day, as smokers replenish nicotine levels depleted during sleep. Time to first cigarette of the day is also used as a proxy for level of nicotine dependence [22]. This cigarette is, therefore, an ideal clinical target for practicing skills for remaining abstinent. It also provides a practical way to standardize practice in a way that all participants can implement and it is simple to assess practice duration. We limited the practice duration to 2 weeks and fMRI assessments to 2, to avoid undue participant burden and to constrain the resources needed for the project within reasonable limits.

Careful consideration was given to the selection of the fMRI task for the proposed study. We selected the smoking cue-reactivity task based on findings supporting robust activations in cognitive control regions (particularly the dlPFC) [23] and based on a wide body of previous neuroimaging studies of smoking showing that cue reactivity can activate the cognitive control networks [18–21, 24].

#### Analysis plan

The planned data analyses are built around three specific aims. The first aim is to compare changes in brain activation in cognitive control regions as a result of cognitive control practice. The second aim is to determine the effect of adherence to the practice regimen on changes in brain activation in cognitive control regions. The third aim is to explore the effects of individual differences (e.g., level of tobacco dependence, motivation, and sex) on changes in brain activation in cognitive control regions.

##### Aim 1

Our primary aim is to identify the impact of practicing delaying the first cigarette of the day compared to not practicing on functional brain activation changes in cognitive control regions. The percentage signal change when comparing the negative smoke condition to the positive smoke condition will be computed for our a priori cognitive control region of interest (ROI) (i.e., dlPFC) for each subject at each time point and entered into a two-way mixed-effects ANOVA model using the group (practice, no practice) × time (pre, post) interaction. We hypothesize that practice vs. no practice will lead to greater pre-to-post brain activation *increases* in the cognitive control network.

##### Aim 2

We will also identify the impact of *adherence* to practicing delaying cigarettes on functional brain activation changes in the cognitive control networks in response to smoking cues. Using only participants from the practice group, we will calculate Pearson correlations between differences in brain activation from the negative smoke and positive smoke conditions and amount of practice completed. Brain activation will be computed as percentage signal change from pre- to post-treatment in response to smoking cues in the a priori dlPFC ROI. Practice will be quantified as the proportion of time delayed from their usual first cigarette of the day to their second cigarette of the day and subjectively measured by participants’ perceived difficulty in delaying the first cigarette of the day. We hypothesize that greater levels of practice will show greater brain activation increases in the cognitive control network in response to negative smoke compared to positive smoke conditions from pre- to post-intervention.

##### Aim 3 (exploratory)

Finally, we will examine the association between practice-related brain activation changes in the cognitive control and reward networks and individual differences (e.g., dependence, motivation, gender, self-report measures of impulsivity, and behavioral measures of cognitive control). We will perform Pearson correlation analyses to examine the relationship between dlPFC brain activation changes (i.e., the percentage signal change in response to negative smoke compared to positive smoke conditions from pre- to post-treatment) and motivation to quit smoking, nicotine dependence, personality and psychological variables, and cognitive performance. In addition, we will use *t*-tests to examine gender differences in brain activation changes. Finally, we will use multiple regression to explore the effects of gender, marijuana use, motivation to quit smoking, dependence, self-report measures of impulsivity, and behavioral measures of executive function, decision-making, inhibition, and demand on changes in cognitive control.

#### Power and sample size

The sample size was based on aim 1 and calculated based on changes in brain activation observed in pilot data following 1 week of practicing CBT skills and 2 weeks of CBT sessions. Assuming the no practice group will have a mean of 0 change in brain activation between day 7 and day 21, and equal variance in the two groups, our preliminary data suggest Cohen’s *d* effect sizes of 2.77 and 3.70 for dlPFC and vmPFC, respectively. These effect sizes suggest *n* = 5 and 4 subjects per group are required to achieve 80% power at 0.05/2 = 0.025 significance level (Bonferroni correction needed to compensate for tests involving each of two brain regions) in a two-sided two-sample *t*-test. However, the small sample in our pilot data (*N* = 6) also indicates wide 95% confidence intervals. Using the conservative estimates of the lower bound of the mean and upper bound of the standard deviation, the effect sizes are (0.0991/0.1415) = 0.70 and (0.2037/0.1889) = 1.08. Using a sample size of *n* = 40 per group provides 79% and > 99% power for dlPFC and vmPFC, respectively; the power will be greater if the actual effect sizes are larger than the conservative estimates.

### Participants

#### Inclusion

Individuals will be eligible if they meet all of the following criteria:

1. Have smoked ≥10 cigarettes per day for the last 6 months
2. Smoke first cigarette within 90 min after waking
3. Age 18 to 60 years
4. Vision normal or corrected to normal (to ensure that they can accurately see the images on the screen and select the appropriate response)
5. Willing to complete all appointments and change smoking behaviors for 2 weeks
6. Have made no quit attempts or attempts to cut back in the last 30 days
7. Have no plans to quit in the next 30 days
8. High school graduate or GED.

#### Exclusion

Individuals will be ineligible if they report any of the following:

1. Serious medical illness unsuitable for the MRI scanner based on best clinical judgment
2. Any history or current symptoms of neurologic or psychiatric disorders except depression, anxiety, or attention-deficit disorder/attention-deficit hyperactivity disorder
3. Currently taking anti-seizure medication
4. History of concussion
5. Body mass index >50
6. Left-handed, due to differences in neural organization between right-handed and left-handed individuals
7. History of alcohol or other substance dependence or current abuse
8. Risk for hazard due to magnetic fields, such as metal in the body due to surgery or an accident (e.g., a pacemaker, cochlear implants, aneurysm clips, intravascular stents or coils, spinal shunt, injury involving bullets, shrapnel or metal implanted in their body, etc.)
9. Pregnancy.

Final eligibility will be adjudicated by the principal investigator.

#### Recruitment

Smokers will be recruited from the Kansas City metropolitan area (population of 2.3 million) using recruitment methods used successfully in the past, including the internet (e.g., Craigslist and Facebook), print, and media advertising (e.g., radio and television spots). Prospective participants will be given a phone number, email address, and website URL to contact for screening. We will also recruit participants from our Clinical Translational Science Award-sponsored Frontiers registry (NIH UL1TR000001), which is a list of smokers who have agreed to be contacted for research studies. All study procedures will take place at the University of Kansas Medical Center, Kansas City, MO, USA.

### Study procedures

#### Consent appointment (day 0)

**Fig. 2:**
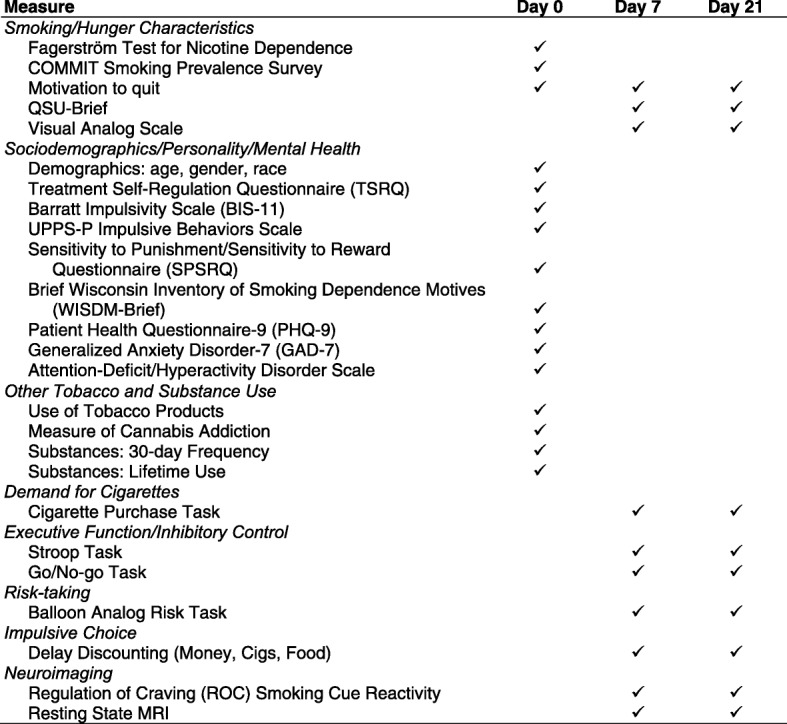
Study measures and timing (SPIRIT Figure). Cigs cigarettes, *MRI* magnetic resonance imaging, *QSU* Questionnaire of Smoking Urges, *UPPS-P* Urgency, Premeditation, Perserverance, Sesation-Seeking, and Positive Urgencey

Study staff will review consent forms describing study goals, procedures, risks, and confidentiality with each participant. Those who consent to participate will be enrolled in the study and given instructions on how to prepare for the baseline session, which will include the first fMRI assessment and daily monitoring of smoking behaviors (days 1–7). In addition, demographic information (including age, gender, education, and income), smoking history, and other data will be collected at the consent appointment. Figure 2 is a full list of questionnaires and tasks administered and the timing of their administration. To promote retention, participants will be compensated for completing questionnaires (Additional file 1).

#### Daily monitoring (days 1–7)

Smoking behaviors will be measured using portable Micro+™ basic Smokerlyzer® CO monitors (coVita, Santa Barbara, CA, USA). The CO monitors come with software that records their output, such as CO levels and a timestamp of each CO measurement. A baseline level will be collected at the beginning of the consent appointment for comparison with levels at the scanning appointments. At this appointment, we will also show participants how to use the CO monitor and instruct them to use it daily during the study. For 1 week following the initial consent appointment, participants will be asked to smoke as usual and provide CO levels by blowing into the CO monitor mouthpiece: (1) upon waking, (2) immediately before smoking the first cigarette of the day, (3) 5 to 10 min following smoking the first cigarette of the day, and (4) immediately before smoking the second cigarette of the day. This will provide timestamps for when participants typically smoke their first and second cigarettes of the day, which will be used as a guide for how long participants in the practice group will be asked to delay smoking their first cigarette.

Participants will also complete paper-and-pencil daily logs recording their first two cigarettes and answering questions relating to their smoking behavior, such as how many cigarettes they smoked that day and if a day was considered typical. Participants will be contacted on days 1 and 3 to see if they are having any difficulty using the monitor and to address any questions. Participants who consent to receiving texts will receive text prompts on days 2 and 4 to remind them to use their CO monitor.

#### Pre-treatment scan appointment (day 7)

Participants will be asked to smoke as usual on the days of testing. They will be given an opportunity to smoke immediately prior to their appointment but not again until they complete testing (about 3–4 h later). This will be done to minimize the dampening effect on cue reactivity of smoking expectancy [18, 25], to control for the time of the last cigarette, and to minimize participant burden. Their height and weight will be measured. There will be at least a 2-h abstinence period prior to the MRI scans. During this time, participants will fill out self-report questionnaires, complete neuropsychological tests, and will be asked to provide a CO measurement. Other studies have used similar abstinence periods and seen expected brain activation patterns to smoking cues [26–31].

Following the fMRI scan, all participants will receive a 10-min CBT style counseling session in which a trained counselor will discuss how to cope with smoking urges, emphasizing problem-solving to overcome difficulties encountered when practicing delaying the first cigarette of the day. We will provide these tips to all participants prior to randomization to control for the influence of the brief counseling on brain activation changes. Without this control, it would be difficult to identify whether brain changes observed in the practice group are due to the brief counseling session or the daily practice of delaying the first cigarette. To promote retention and completion of daily questionnaires, participants will be compensated at the second visit for their time, for completing the one week of CO measurements

#### Randomization and blinding

Following the brief counseling, participants will be randomized using a 1:1 allocation ratio to the practice or no practice arm of the study. Groups will be stratified on gender and marijuana use to prevent these variables from confounding treatment effects. Based on previous recruitment data, we expect a 1:1 ratio of men to women and a 1:2 ratio of marijuana users to non-marijuana users. Separate blocked randomization sequences (www.randomization.com) for each of the four strata will be used. A person from outside the study team, who will have no interaction with the participants or the data, will generate the sequence and reveal the group assignments prior to building the practice schedule. Staff responsible for enrolling subjects will not participate in generating the randomization sequence and will not have specific knowledge of how it was generated.

Study staff who interact with participants will be blinded to group membership until after the brief counseling session. They will not be informed of group membership until it is time to reveal it to the participants. Participants will not be blinded to group membership because there is no way to institute the intervention without revealing group membership.

#### Intervention (days 8–21)

For 2 weeks following the initial fMRI appointment, CO levels will be recorded daily by having the participant blow into the CO monitor mouthpiece upon waking, immediately before smoking the first cigarette of the day, 5 to 10 min following smoking the first cigarette of the day, and immediately before smoking the second cigarette of the day. This will provide data on adherence to delaying or not delaying the first cigarette of the day as instructed. Participants will receive a follow-up phone call or text on day 8 to see how the use of the monitor is working and/or how practicing delaying the first cigarette of the day is going. In addition, we will address any participant questions. Participants who consent to receiving texts will also receive text prompts on days 9, 11, 16, and 18 to remind them to use their CO monitor and/or ask how practicing delaying the first cigarette of the day is going.

##### Practice group

Half of the participants will be randomized to the practice group. Data from self-reported typical days from the initial week of monitoring will be averaged to build participants’ schedules (Table 1). To standardize practice for our experiments, we will ask smokers to delay their first cigarette of the day on a progressive schedule for 1 week with the goal of delaying their first cigarette to the time of their usual second cigarette of the day or later. The maximum delay will be maintained during the second week for a total of 2 weeks of practice. On day 14, practice group participants will be sent a text asking, “On a scale from 1 to 5: 1 = not at all difficult and 5 = extremely difficult – how difficult would you say delaying your first cigarette has been the past two days?” If participants report a 1 or 5 for either day, they will be sent an adjusted schedule. In the case of a 1, the following 2 days will continue the progressive schedule until the delay is 2.33 times the length of the normal delay. In the case of a 5, participants will be instructed to revert to the day 12 delay (1.33 times the normal delay) for the remainder of the practice days. Participants will continue to fill out the paper-and-pencil daily monitoring log with the addition of questions about how difficult it was to delay the time to the first cigarette of the day on a scale of 1 = not at all difficult to 5 = extremely difficult and what strategies they used to delay the time to the first cigarette.

**Table 1:** Examples of cognitive control regimens for the practice group

	Delay to 1st cigarette calculation	Example for smoker who smokes 1st cigarette of the day 5 min after waking and 2nd cigarette of the day 60 min later	Example for smoker who smokes 1st cigarette of the day 30 min after waking and 2nd cigarette of the day 90 min later
Baseline week (days 1–7)	Usual time	5 min	30 min
Day 8	Usual time + 0.33 of usual time to 2nd cigarette	~ 25 min	~ 60 min
Day 9	Usual time + 0.5 of usual time to 2nd cigarette	~ 35 min	~ 75 min
Day 10	Usual time + 0.66 of usual time to 2nd cigarette	~ 45 min	~ 90 min
Day 11	Usual time + 1.00 of usual time to 2nd cigarette	~ 65 min	~ 120 min
Day 12	Usual time + 1.33 of usual time to 2nd cigarette	~ 85 min	~ 150 min
Day 13	Usual time + 1.5 of usual time to 2nd cigarette	~ 95 min	~ 165 min
Days 14–20	Usual time + 1.66 of usual time to 2nd cigarette	~ 105 min	~ 180 min
Day 21 (follow-up fMRI)	Usual time	5 min	30 min

*fMRI* functional magnetic resonance imaging

##### No practice group

Participants in the no practice arm will not be instructed to change their smoking behavior in any way. Instead these participants will be instructed to smoke their cigarettes as usual over a 2-week period and to continue monitoring their smoking using the CO monitor and daily monitoring logs so that the study procedures for both arms are as similar as possible. The daily monitoring log will have an additional question asking whether they used CBT techniques at other times during the day to change or reduce their smoking. We chose a monitoring-only control arm to isolate the impact on brain activation of practicing skills following brief advice compared to any disruption in usual smoking behaviors engendered by monitoring itself [32].

#### Post-treatment scan appointment (day 21)

The post-treatment scan appointment will include the same self-report, behavioral, and fMRI procedures as the pre-treatment scan appointment (see Fig. 2). In addition, participants will complete an end-of-study assessment and debriefing including open-ended questions about the difficulty of the intervention, study experiences, and behavioral techniques employed to delay smoking the first cigarette of the day. Participants will also be given an overview of the goals of the study and anticipated outcomes to encourage engagement in research studies in the future. To promote retention and completion of daily questionnaires, participants will be compensated at the second visit for their time, for completing the 2 weeks of CO measurements (i.e., double the potential payment for the first week of measurements), and for returning the CO monitor.

### Measures

#### fMRI assessments

##### Image acquisition

Scanning will be performed on a 3-tesla Siemens Skyra scanner (Siemens, Erlangen, Germany) fitted with a 20-channel head and neck coil. Following automated scout image acquisition and shimming procedures to optimize field homogeneity, resting state, fMRI task, and structural scans will be acquired. Resting state scanning parameters will include BOLD sequences of 52 contiguous slices at a 40° angle to the anterior commissure–posterior commissure (AC-PC) line (repetition time/echo time [time repetition (TR)/time echo (TE)] = 3000/25 ms, flip angle = 90°, field of view = 640 × 640, matrix = 80 × 80, slice thickness = 3 mm, in plane resolution = 2.9 mm, 200 data points). Task scanning parameters will include gradient echo BOLD scans (five, one for each run of the craving rating task), which will be acquired in 43 contiguous oblique axial slices at a 40° angle (TR/TE = 2500/25 ms, flip angle = 90°, field of view = 560 × 560 mm, matrix = 80 × 80, slice thickness = 3.0 mm, in-plane resolution = 2.9 × 2.9 mm, 145 data points). Finally, a T1-weighted structural scan will be completed (3D MPRAGE sequence, TR/TE = 2300/2.95 ms, flip angle = 9°, field of view = 253 × 270 mm, matrix = 240 × 256, slice thickness = 1.2 mm, 176 slices). This scan will be used for Talairach transformation and coregistration with fMRI data. All participants will be positioned in the scanner so that the angle of the AC-PC plane is between 17° and 22° to the scanner coordinate space, ensuring that the 40° slice acquisition angle is constant for all participants. Preprocessing of resting and task fMRI data will be performed for each participant in AFNI (Medical College of Wisconsin).

##### Regulation-of-craving cognitive control task

**Fig. 3:**
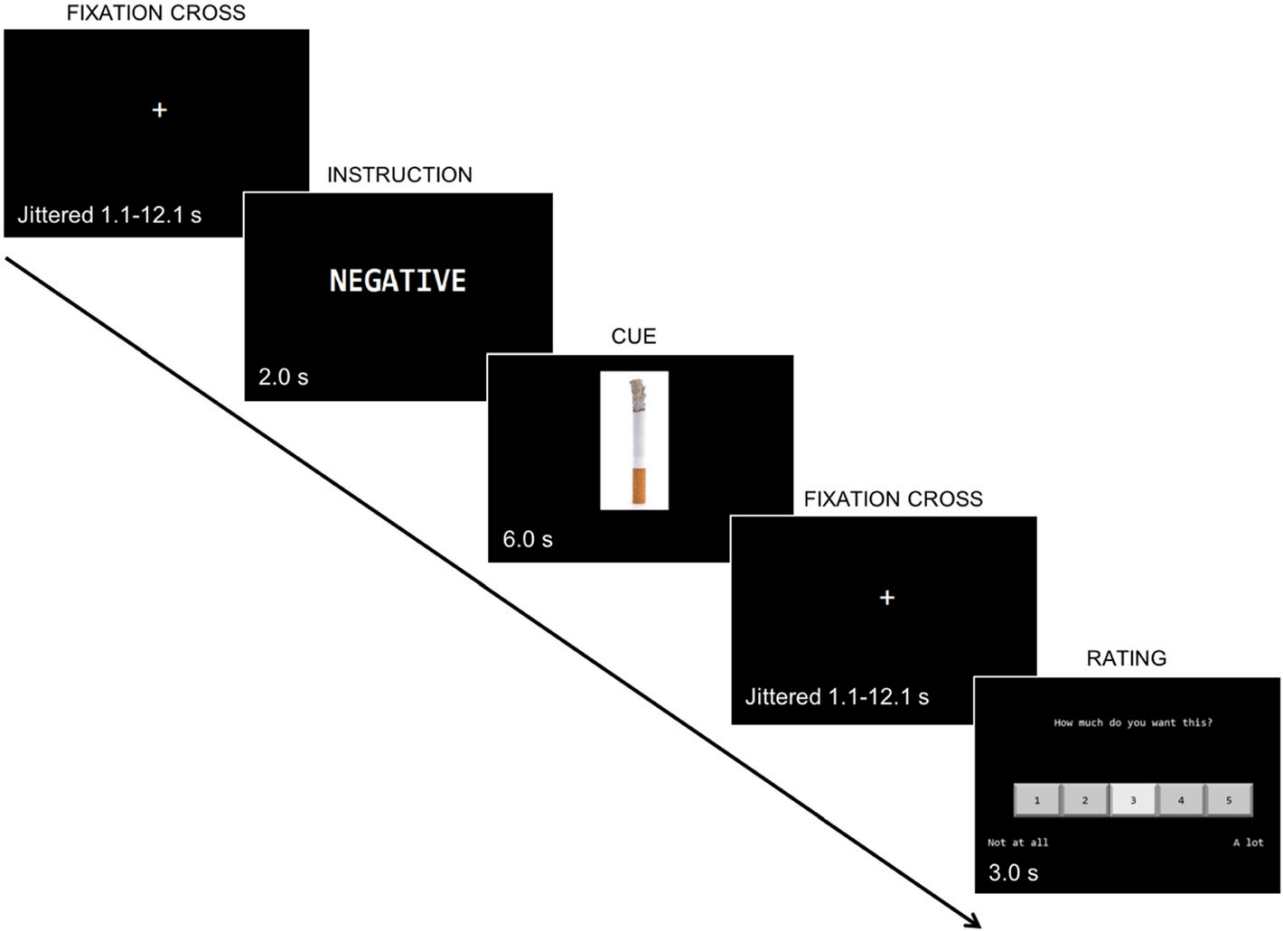
Diagram depicting scanner task

During the ~ 30-min fMRI scan, participants will complete a regulation-of-craving task closely modeled after Kober et al. [23] in which they will view smoking and food cues and will be asked to rate “How much do you want this?” the items on a scale of 1 to 5 (1 = not at all; 5 = a lot) after viewing them (see Fig. 3). Smoking cues will include pictures such as people smoking and packs of cigarettes. Food cues will include pictures of appetizing food items such as pizza. All cues have been acquired from previous smoking and food cue reactivity studies [20, 23, 33, 34] and stock photography websites. Participants will receive instructions to regulate their responses to these images at the start of each trial. Half of the trials will be positive trials and half will be negative trials. In positive trials, participants will be instructed to think about the positive consequences of consuming the presented item. In negative trials, participants will be instructed to think about the negative consequences of consuming the presented item. A practice session will be conducted outside the scanner immediately prior to the scan to ensure that the participants understand the task. Following the imaging session, participants will be asked to describe the strategies they used during the task. Scanning will be done in five runs of 20 trials each (100 total trials), with trials of each of the four possible types—positive smoking, positive food, negative smoking, and negative food—presented in pseudorandom order. Participants will see different stimuli sets at each MRI appointment, which will be counterbalanced across participants. Stimuli will be presented using E-Prime 3 (Psychology Software Tools). Optimal timing of stimuli will be estimated using the Analysis of Functional Neuroimages (AFNI) stimulus timing program (make_random_timing.py).

##### Regulation-of-craving task data preprocessing

The fMRI images will be realigned to the third slice collected in each scanning session to correct for motion. The images will be spatially smoothed with a 4-mm full width at half maximum (FWHM) Gaussian blur. Timepoints during which participants move more than 0.3 mm within a TR will be censored. Functional and anatomic images obtained within each session will be aligned and normalized to Talairach and Tournoux’s [35] stereotaxic atlas. Regressors representing the experimental conditions of interest (i.e., positive smoke, negative smoke, positive food, and negative food) will be modeled with a block response function and entered into the multiple-regression analysis using a random-effects model. Motion parameters will be included as nuisance regressors.

The primary contrast of interest will be negative smoke minus positive smoke, which represents an analog of the exertion of cognitive control minus general brain activation engendered by attending to smoking cues. The primary outcome analysis will focus on the a priori ROI, the dlPFC. In addition, an exploratory whole-brain analysis will be used to identify regions outside the dlPFC cognitive control network that may change with practice (e.g., the insula). The dlPFC ROI has been selected based on previous studies [5, 23, 24, 36]. The dlPFC will be defined using anatomical masks for the dlPFC based on the superior frontal gyrus and the middle frontal gyrus regions identified by AFNI’s whereami function. Multiple comparisons will be corrected using a familywise approach in which clusters will be considered significant at *p*_voxelwise_ < .01 and *p*_corrected_ < .05 within the dlPFC for the ROI analysis and within the whole-brain for the exploratory whole-brain analysis. The minimum cluster size will be determined using AFNI’s 3dClustSim function. The spatial autocorrelation function (acf) option was used in AFNI’s 3dFWHMx to estimate intrinsic smoothness and 3dClustSim to estimate the probability of false positives [37, 38]. The mean percentage signal change from dlPFC clusters that pass cluster thresholding will be used as the primary outcome data to test the hypothesis described in aim 1.

##### Resting state scan

Prior to completing the regulation-of-craving task, approximately 10 min of resting state data will be acquired while participants are instructed to keep their eyes open and focused on a fixation cross at the center of the screen. Heart rate and respiration rate will be collected using Biopac (BIOPAC MP150 Data Acquisition). This scan will be used in exploratory analyses to examine changes in resting state connectivity between cognitive control regions and the rest of the brain following the intervention*.*

##### Resting state data preprocessing

Freesurfer will be used to segment gray matter, white matter, and cerebral spinal fluid. In addition, respiration and heart rate will be processed using AFNI’s RetroTS. Preprocessing analysis scripts will be built using afni_restproc.py [39]. Preprocessing will include removing the first four volumes, removing any transient signal, slice time correction, and co-registering all functional data to the first volume. Nuisance variables will be measured [i.e., six motion parameters (three translations and three rotations), average ventricle signal and average local white matter signal (15-mm spherical neighborhood, 3dLocalstat)] and removed from the signal time course using multiple regression. The residual time course images will then be smoothed with a 6-mm FWHM Gaussian kernel, resampled to a 2 × 2 × 2 mm grid, and spatially normalized to Talairach stereotaxic space. In addition to controlling for six motion parameters, further motion correction procedures (i.e., scrubbing) will be used to reduce the possibility of type I errors related to motion [40, 41]. Time points with greater than 0.3-mm motion will be censored.

#### Self-report assessments

##### Smoking characteristics

The Fagerström Test for Nicotine Dependence [42] will assess nicotine dependence. Items from the COMMIT Smoking Prevalence Survey [43] will assess smoking history such as tobacco use (cigarettes per day (CPD) and other tobacco use), age when first smoked, and quitting/relapse history. Because neural responses may be influenced by motivation to quit, we will assess this by asking: “How motivated are you to quit smoking?” on a scale from 0 to 10.

##### Individual differences in personality, smoking attitudes, and mood

Autonomous regulation for smoking cessation will be assessed using the Treatment Self-Regulation Questionnaire [44], an established measure of internalized motivation for behavior change. Self-report impulsivity will be measured with the Barratt Impulsivity Scale (BIS-11) [45] and the UPPS-P Impulsive Behavior Scale [46]. Sensitivity to reward will be measured with the Sensitivity to Punishment/Sensitivity to Reward Questionnaire (SPSRQ) [47]. Items from the multidimensional Brief Wisconsin Inventory of Smoking Dependence Motives [48] will assess primary and secondary dependence motives. The Patient Health Questionnaire-9 (PHQ-9) [49], Generalized Anxiety Disorder-7 scale (GAD-7) [50], and Attention-Deficit/Hyperactivity Disorder (ADHD) Symptoms scale (from the PhenX Toolkit [51]) will assess depression, anxiety, and ADHD symptoms, respectively.

##### Other tobacco and substance use

Participants will answer questions on cannabis addiction, which focuses on the diagnostic criteria for cannabis use disorder. To create this measure, we adapted questions from the Yale Food Addiction Scale [52], which assesses multiple aspects of addiction, such as tolerance, frequency of use, and preoccupation. Other tobacco and substance use will be characterized using the following instruments from the PhenX Toolkit [51]: Use of Tobacco Products, Substances: 30-day Frequency, Substances: Lifetime Use.

#### Behavioral measures

Measures of the demand for cigarettes, executive function/inhibitory control, risk-taking, and impulsive choice will be administered outside of the scanner to test if practice leads to behavioral changes.

##### Demand for cigarettes

A cigarette purchase task [53, 54] will be used to measure the demand for cigarettes. In this task, participants are asked how many cigarettes they would smoke in a day across a wide range of hypothetical per-cigarette prices ($0.01 to $35). The resulting data will be modeled to yield a number of economic indicators related to demand including elasticity, intensity, breakpoint, and maximum expenditure.

##### Executive function and inhibitory control

A Stroop task and a go/no-go task will be used as measures of executive function/inhibitory control. In the Stroop task [55], participants are asked to press keyboard buttons corresponding to the font color of neutral words (e.g., “when” and “and”) and color words (e.g., “blue” and “red”) presented on-screen using the Psychology Experiment Building Language (PEBL) [56]. In some trials (incongruent trials), the color words are presented in a mismatching font color (e.g., the word “yellow” in a green font). In these cases, participants must ignore the semantic content of the word to get the correct answer. Errors and the response times in these trials (compared to the congruent trials) will be used as the primary variables.

In the go/no-go task [57], participants consecutively see individual letters on a computer monitor. This test was developed using E-Prime 3.0 (Psychology Software Tools, Inc., Sharpsburg, PA, USA). Participants are asked to press a button if and only if a certain sequence of letters occurs (e.g., “S” followed by “T”). These are considered go trials. When “S” is followed by any other letter, it is considered a no-go trial. Several measures will be computed including the percentage of correct go responses, mean reaction time of go responses, percentage of false alarms (overall and just on no-go trials), and impulsivity and inattention scores as defined by Overtoom et al. [58].

##### Risk-taking

The PEBL version of the balloon analog risk task [59, 60] will be used to assess risk-taking. Participants will be asked to press a button that pumps up an on-screen balloon. Every pump is rewarded with a small amount of virtual money but carries the risk of bursting the balloon. Participants may choose to bank the money earned for a balloon at any time, thus ending the trial. Popping the balloon resets the money counter back to zero and starts a new trial. The number of popped balloons and the total banked money on non-popped trials will be used as primary measures.

##### Impulsive choice

A computerized delay discounting task [61, 62] (E-Prime 3.0) will be used to assess impulsive choice. The delay discounting task asks participants to choose between hypothetical smaller-sooner and larger-later rewards across a wide range of delays (1 day to 25 years). The smaller reward is adjusted after each choice (up if the participant rejected it on the previous trial or down if the participant accepted it) and the value of the smaller reward after five choices is taken as the indifference point or switch point. Participants will complete three versions of the task, each with a different reward type (money, cigarettes, or food) roughly equated for value. The extent to which each commodity loses value as a function of time-to-receipt, determined by mathematical modeling of the discounting curve created by plotting switch points as a function of delay, will be the primary measure of impulsivity.

### Data management

Study data will be collected and managed using REDCap (Research Electronic Data Capture), which is hosted at the University of Kansas Medical Center [63]. REDCap is a secure web-based application designed to support data capture for research studies. It provides: (1) an intuitive interface for validated data entry, (2) an audit trail for tracking data manipulation and export procedures, (3) automated export procedures for seamless data downloads to common statistical packages, and (4) procedures for importing data from external sources. All self-report data will be entered directly into REDCap by participants and all daily monitoring logs will be double entered. Physical documents will be kept in a locked cabinet accessible only by study staff to comply with confidentiality requirements. MRI data will be sent directly from the scanner to the Hoglund Brain Imaging Center server, which runs XNAT 1.6.4 [64], an open-source imaging informatics platform designed to facilitate management of imaging. Task and resting state fMRI data will be checked by trained research personnel to ensure the quality of acquired and preprocessed data prior to group analysis. As a relatively short-term study with minimal participant risks, a data monitoring committee will not be used. Any adverse events (although these are anticipated to be minimal) will be reported to the local institutional review board as per local standards. The trial will be subject to random auditing per local standards but no auditing by within-study staff is planned.

### Modifications of the protocol

Any modifications to the protocol that may impact on the conduct of the study, the potential benefits to the patients, or patient safety, including changes of study objectives, study design, population, sample sizes, study procedures, or significant administrative aspects, will require a formal amendment to the protocol. Such amendments will be at the discretion of the principal investigator in consultation with other key study personnel. Any such major changes will be communicated to ClinicalTrials.gov.

## Discussion

Despite advances in treatments for smoking cessation, most quit attempts fail and smoking still places an unacceptable burden on public health. Nonpharmacological treatment is somewhat effective but the precise mechanism of how it works remains mostly unknown. Dual models of addiction suggest that any effective treatment of addiction should target the neural pathways involved in cognitive control [5]. We are testing the overarching hypothesis that explicit cognitive control-related practice related to smoking will increase smokers cognitive control capacity, which will be reflected in cognitive control-related brain activation.

When complete, this project is expected to achieve two goals. First, we are beginning the process of linking treatment research with the extensive work on developing neural models of addiction. The value of neural models of addiction can only be realized if they are used to understand and inform treatment approaches. The dual-system models of addiction suggest that treatments that enhance cognitive control should help smokers to quit more successfully. Whether existing treatments have this effect is unknown. Whether existing treatments can be optimized or targeted to appropriate individuals to maximize the neural impact is unknown. Second, establishing important biomarkers of treatment effects could lead to efficient and powerful ways of pre-testing or optimizing novel treatments prior to conducting large expensive randomized clinical trials. This project could serve as a launching point for many similar experiments to determine which interventions are worth scaling up to larger more involved experimental designs.

### Trial Status

Recruitment began in December 2016 and is planned to conclude in June 2019. The current protocol has been approved by the University of Kansas Medical Center Human Subjects Committee, ID STUDY00004095, version 10.01.

## Additional file

Additional file 1: SPIRIT 2013 Checklist: Recommended items to address in a clinical trial protocol and related documents. (DOC 121 kb)
